# The Fall and Rise of Racial Inequality in London Homicides: a Challenge for Policing by Consent

**DOI:** 10.1007/s41887-022-00084-9

**Published:** 2022-12-07

**Authors:** Sumit Kumar, Lawrence W. Sherman, Heather Strang

**Affiliations:** 1 Cambridge Centre for Evidence-Based Policing, Cambridge, UK; 2 Institute of Criminology, University of Cambridge and Metropolitan Police Service, London, UK; 3 Institute of Criminology, University of Cambridge, Cambridge, UK

**Keywords:** Homicide, Inequality, Race, Ethnicity, Age, Gender, Crime reduction, Policing

## Abstract

**Research Question:**

How have London’s racial and demographic disparities in homicide victimisation rates changed in 2 decades of the twenty-first century, with what implications for policing by consent?

**Data:**

We collected Metropolitan Police Service homicide victimisation counts in London for each financial year (April through March) so far in the twenty-first century, by race, gender and age. We also collected the estimated residential population size of those groups from the 2001 and 2011 Census results.

**Methods:**

We divided the number of homicides each year in each demographic category by the estimated population size of that category, and then computed victimisation rates per 100,000 for each of the 21 years. We plotted trends in the rates of each group over time, whilst calculating ratios between victimisation rates of Blacks and Whites, and of South Asians and Whites, in each year.

**Findings:**

Over the past 2 decades in London, Black homicide victimisation rates have fallen by almost half, but they remain about 5 times higher than homicide victimisations of Whites and South Asians. Inequality of homicide rates between Black and White victimisations declined substantially, but then became worse: the most recent 5 years showed 19% more inequality than in the century’s first 5 years. Three major changes in homicide inequalities have occurred since 2001: (A) The total Black homicide victimisation rate dropped by 71% from 2001 to 2014; (B) homicides of Blacks then increased by 92% in the 5 years to 2019–2020, whilst the White victimisation rate remained unchanged; (C) from 2019 to 2022, Black victimisation rates declined again by 27%, whilst White rates also declined, by 26%. Young Black *males* aged 16–24 were 10 to 20 times more likely than White counterparts to become homicide victims in 2017–2022. Yet Black *female* homicide victimisation dropped by 82% over 21 years. Female inequality reduced from up to 400% higher for Black females than Whites at the beginning of the century to 67% higher in the most recent 5 years. For Asians of all ages, inequality of homicide victimisations to Whites disappeared by 2022. Inequality persisted between young Asian males and young White males.

**Conclusion:**

Changes in London’s racial inequality in homicide victimisation are both substantial and volatile. Understanding their fall and rise may help police to renew and sustain reductions of racial inequality in risks of violence. Learning lessons about what police may have done to cause substantial reductions in Black victimisation requires both retrospective and ongoing tracking of both homicide and policing at local levels. Providing transparent tracking is also essential to public dialogue about policing strategies, which could help to renew policing by consent based on precise statistical evidence.

## Introduction

“Policing by consent” is a bedrock principle of British policing. Echoing the phrase “consent of the governed” in the 1776 US Declaration of Independence, policing by consent is tied by police historians to the nine “Peelian Principles” prescribed around the 1829 founding of London’s Metropolitan Police (Lentz & Chaires, 2007). More recently, the UK Home Office (2012) has defined this concept as an idea that.

refers to the power of the police coming from the common consent of the public, as opposed to the power of the state. It does not mean the consent of an individual. No individual can choose to withdraw his or her consent from the police, or from a law.

Putting this idea into operational practice has long been a challenge for police leaders, who have developed many responses. One response has been to *tell* people what police are doing to protect them. More recently, a second response has developed of *asking* people what their priorities are for policing in their neighbourhoods (Skogan, 2004). More recent still is the idea of police engaging in a *dialogue* about what police should do in their community, and how well they are doing it (Bottoms & Tankebe, 2012). Whilst the specific features of this “dialogic” approach to policing by consent have not been developed through testing in real-life settings, the concept offers a major opportunity for evidence-based policing.

In contrast to asking—and listening to—people about their concerns (Skogan, 2004), a dialogue can begin with police presenting evidence to community residents and leaders, and a choice of possible police actions in response to that evidence. A detailed statement of the facts of crime and safety by police could then be followed by responses from a wide range of community members. Those responses could lead to further points, or even more evidence, to be supplied by police. The evidence in question could be about local crime patterns (such as the 32 boroughs or 4835 Lower Super Output Areas of London), or even patterns found across the entire police jurisdiction (such as all of London).

The substance of the evidence about severity or frequency of crime problems could frame a discussion, in which police may propose to offer more or less policing (or intrusiveness of police tactics), depending on the level of harm each community suffers. These discussions could focus on a range of issues that appear in metrics of public perceptions of what police do and how well they do it in different communities. Yet because such discussions do not routinely provide current data on crime and policing, it is no surprise that even when discussions occur, the public may find them unsatisfactory. Repeated surveys may indicate various levels of dissatisfaction for various reasons. But where they are in decline, there is every reason to try a new approach—such as providing more detailed evidence to the public.

### Consent in London: an 8-Year Decline

One major example of public satisfaction in decline has been the closely measured tracking of opinions of London residents from December 2014 through March 2022. The public surveys found on the website of the London Mayor’s Office of Police and Crime (MOPAC, 2022) report trends on a number of measures of police performance. The consistent result across multiple indicators is downwards. Over 8 years, the percentage of affirmative answers to the following questions dropped by the percentages indicated below:

**Table:** 

Question	Percent yes in 2014	Percent yes in 2022
Informed: *Well-informed about police activities*	49%	38%
Listen: *Police listen to concerns of local people*	74%	64%
Fair: *Police treat everyone fairly no matter who they are*	74%	62%
Good job: *Police do a good job in the local area*	57%	49%
Matter: *Police are dealing with things that matter to this community*	72%	60%

Whether these findings depend heavily on what happens in community meetings is a matter of some speculation. No matter how many people attend—in person or on a video-conference call—they will still comprise a tiny fraction of the local population. And no matter how many other people they tell about what police said (or did not say) in a meeting, the proportion of residents who hear anything about it will still be low. What is more plausible, perhaps, is that their views of the police may be shaped more by “signal crimes” (Innes, 2004), defined as crimes which attract substantial attention and cause emotional disturbance in the community. Their views may also be affected by media attention to what police do elsewhere, even when it happens in other countries (Laniyonu, 2022). Thus, any strategy that aims primarily at better information to discuss in police-community meetings seems unlikely to raise public confidence in police.

Yet if the realities of crime and policing in their own community are hypothesised to be a key factor in policing by consent, there may be better evidence. “Crime and policing” consist of thousands of events across London every day. Each event has ripple effects of pain, or relief, suffering or gratitude, all of which may shape public perceptions. Moreover, those events may be perceived differently because of underlying social conditions—things that affect health, wealth, education and life outcomes. Foremost amongst these, by some evidence, may be a multidimensional concept of *inequality*, shaped as much by historical and structural forces as by what the police did yesterday. For all those reasons, better police tracking of inequalities in crime may not only help promote dialogue with communities. It may also promote dialogue within police agencies about what *kind* of policing officers should propose to communities and test as a means of reducing both crime and inequality itself.

### Inequality and Consent

Inequality is a central issue for policing by consent, and all consent of the governed, in modern democracies. Many reasons have been suggested for this fact—including the strong association of inequality with death rates from all causes (Marmot, 2020). In attempts to predict homicide, criminologists have consistently found strong associations between murder rates and inequality measures at national, regional and local levels of analysis (Daly, 2017). Yet, in the world of policing and local politics, homicide inequality receives a relatively little public comment.

Inequalities in criminal victimisation pose a fundamental challenge for the police duty to provide equal protection. Even if police drive down *total* crime and violence, the result may be seen as an unsatisfactory if racial and demographic inequality of victimisation persists. Yet if *inequality* of victimisation declines, it is essential for police to know (a) that it has happened, and (b) what, if anything, police did to reduce inequality. All of this requires precision tracking of racial and demographic inequalities over time in *criminal victimisation* (as distinct from *policing*). Yet precision tracking of this kind has been missing in action from most, if not all, police agencies in liberal democracies.

There are many potential consequences of providing evidence on inequality in criminal victimisation rates per capita. At the very least, the evidence raises questions (and demands) for evidence on police resources. Whether residents perceive their areas as over-policed or under-policed may be a question that provokes a different answer in the context of evidence on inequality. It may also have profound consequences for the level of trust and confidence residents have in their local police, as in the hypothesis implied by the dialogic approach (Bottoms & Tankebe, 2012).

### Homicide Inequality as a Bellwether for Unequal Crime Victimisation Rates

The clearest example of this gap is a lack of official reporting of the statistical rates at which different demographic groups become victims of homicide. That gap is unimaginable in related fields, such as public health. The death rates from COVID-19, for example, were demographically disaggregated by leading statisticians and the UK Office of National Statistics from the very start of the Pandemic. Yet we can find no evidence of any UK or US police agency routinely reporting its own population’s demographically disaggregated rates of death from homicide—let alone trends in disparities over time.

In the first days of COVID, before vaccines were available, COVID mortality for Black males was about three times higher than COVID deaths for While males (ONS, 2022), a fact that was widely reported to great concern. Yet in the past decade, homicide mortality for young Black males in London was thirteen higher than for young White males (see Fig. 5 below). But for many police leaders, close tracking of racial inequalities in homicide victimisation would amount to “false precision”. Police already have “good enough” knowledge to do their job, they could say (Sherman, 2022a). All they need to know is that young Black men are murdered at much higher rates than young White males, or any other demographic category. Police can then apply that knowledge to allocate proportionately more resources to the prevention of violent crime in the highest risk group. Yet if the public do not agree that extra policing or certain tactics are needed in their area, it may be that police need to share much more precise knowledge of differential risk levels with the communities themselves.

Policing by consent requires that communities, and not just police leaders, see and understand differential policing as *proportionate* to a racial disparity in victimisation that was not caused by policing itself. One recent critique in the USA, in fact, claims that police have caused higher violence rates against minority groups by providing *fewer* police officers per capita to prevent crimes against Blacks than to prevent crimes against Whites (Lewis & Usmani, 2022).

As for preventive policing tactics, such as stop and search, focused on removing illegal weapons from public places, there is a clear need to demonstrate the evidence for high risk of such crimes in a few “red zones” around a city, accompanied by “amber” and “green” zones where stop and search has no evidence of preventing violence. As Gladwell (2019) observes, the use of stop-search tactics in very high violence areas may be essential to preventing weapon murders. But using the tactic anywhere that has no violent crime—such as most parts of most communities—is a clear example of over-policing unlikely to attract public consent.

Here are four reasons for doing what this report offers, on a regular basis, in every police agency in which murders regularly occur, by way of *tracking* the key dimension of racial inequality in homicide victimisations.

1. If the public are *not* conscious of how much higher rates of homicide are against some ethnic or demographic groups, they may well withhold their consent for extra policing distributed amongst members of those groups. Without adequate demonstration of increased risk of murder, they may find extra policing to be disproportionate: “over-policing” rather than “under-policing” (Sherman, 2022b). Absent clear evidence of the need for additional intrusions into public liberty in different areas with unequal rates of violence, police may be criticised for unequal policing, rather than for a failure to provide equal protection against unequal risk (Lewis & Usmani, 2022; Sherman & Kumar, 2021).
2. If the precise magnitudes of racial disparities are not tracked over time, it is impossible to tell whether the problem of *inequality* (as distinct from homicide) is getting worse or better, and by how much. Police legitimacy suffers not just because of high crime against minorities, but from the perceived unfairness of higher crime *relative* to majority groups. The demand for *equal* outcomes may be more important for legitimacy than for *better* outcomes. Reducing homicides overall can actually increase racial disparity in homicides, and hence decrease perceptions of trust and confidence in police to be fair.
3. If the disparities in violence are not targeted and tracked by micro-geographic areas (such as “hot spots” of violence), there is no way to tell if differences in police practices by location are correlated with differences in racial disparity trends (see Sherman (2013), on targeting, testing and tracking for evidence-based policing). Whilst police may target more young Black men to protect them, intrusive tactics in *low-crime* areas may be useless as well as infuriating to those targeted. Adjusting proactive tactics to the victimisation rates by race requires tracking those rates with a micro-level focus (Sherman & Kumar, 2021).
4. If the rates of homicide go down in a high-homicide demographic group, that fact can be hailed mistakenly as a drop in racial inequality (see point #2 above). Since the homicide rate can also go down in the lower-homicide group as well, there may be little or no change in inequality.

### Public Dialogue and Inequalities

One way to address the question of policing by consent in reducing racial inequalities in victimisation is to offer more precise evidence for *public dialogue*, as a means of promoting police legitimacy. As Bottoms and Tankebe (2012) have theorised, police legitimacy must be sustained in a constant dialogue about what police are doing, what they are *not* doing, and why. These dialogues can take place in face-to-face community meetings, in online discussion platforms such as Zoom, in hybrid meetings with police or in public hearings with local legislators. No matter what the medium of a discussion, the central importance of clear statistical facts remains. Police themselves would need to have these facts before presenting them at public meetings. Tracking these facts and discussing them seem to be a prerequisite for “dialogic legitimacy”.

The purpose of this report is to demonstrate what can be learned by tracking inequalities of criminal offences against majority and minority groups in one major metropolis. That purpose requires a long enough period of observation to be able to put recent trends into perspective. That longer perspective is especially important for relatively rare kinds of crime, such as homicide. This kind of analysis is essential to identify, in retrospect, what police may have done to reduce or increase racial inequalities in the risk of homicide. The case of London provides just such an opportunity.

## Research Question

The context for our research question offers relatively little prior research to offer benchmarks of inequality, even in official statistics. Precisely how big a difference there is in homicide victimisation rates by race has received little attention in even the most advanced economies. National multi-year trends in these differences, for example, are not reported in the UK, Canada, New Zealand or the EU. They are tracked in the USA, however, by the Center for Disease Control’s National Center for Health Statistics (Quickstats 2017), which reported that, across all age and gender groups, Black Americans were about 7 times more likely than Whites to be homicide victims in 1999–2015.

National statistics on racial disparities, of course, may be very different from what could be reported by local policing. Inequalities may vary widely within the same country. At the level of each territorial police agency, a precise ratio of homicide victimisation rates by race is a number even more rarely reported, let alone discussed in police-community dialogues. If the value of tracking these indicators of extreme violence can be demonstrated in one highly visible police force, perhaps many others would follow that lead.

London offers several advantages as a demonstration site for calculating and tracking racial disparities in homicide victimisations. One is that the overall homicide rate in London (1.4 per 100,000 in 2021–2022) is relatively low for a global city, in comparison, for example, to substantially higher rates per 100,000 in Madrid (12.0) and New York (5.5).^1^ A second is that London is highly diverse, with a reported 2019 breakdown of London’s population as 54% White, 20% Black and 19% Asian (London Councils, 2022). A third is that ethnic disparity in homicide rates is widely assumed based on case-by-case reporting in the news media of about 2 to 3 homicides per week. If a diverse population can experience substantial ethnic inequalities in homicide even in a low-homicide rate city, that fact could provide evidence that such disparities are important to track—and to notice when they get worse or better.

We, therefore, frame the research question for this exploratory research note as follows:

How have London’s racial and demographic disparities in homicide victimisation changed in the twenty-first century, with what implications for policing by consent?

## Data

The Metropolitan Police Service (MPS) assisted us in answering this question by providing the data required. Officers and staff of the Strategic Insight Unit extracted the reported demographic characteristics of victims in all homicide reports from April 2000 through March 2022 from the CRIS database (Crime Records Information System). Throughout the entire study period, CRIS was the only database used by MPS to record all crime reports. Whatever flaws may have been inherent in the system were unlikely to change over the time period. The result is that these data should be fairly reliable, although with some items of missing or inaccurate data possible across several thousand reports.

The four categories of an ethnic group that we aggregated for analysis are as follows:

- White: both British and non-British combined (including Europeans)
- Black: both British and non-British combined, including all those of African, Afro-Caribbean, Afro-American or other multi-origin categories.
- Asian: *South* Asians from Indian Subcontinent (India, Pakistan, Bangladesh, Sri Lanka)
- All others: Chinese, Japanese, Southeast Asian, Arabic or North African, or others not in categories above.

For the purposes of this exploratory analysis, we concentrated on the three largest groups: White, Black and [South] Asian. Homicide victims of all other national origins were excluded, solely on the basis of limiting the present study to an exploratory demonstration.

We also used CRIS records on the sex and age of each victim, which increased our capacity to demonstrate large variations in risk of being killed by homicide across these other demographic categories—a well-established fact in the global literature on criminal violence (e.g. Reiss & Roth, 1993).

As we wrote in our previous analysis across England and Wales (Kumar et al., 2020: 180):

We also collected the estimated population size of those groups from the 2001 and 2011 Census. To the extent possible, we tried to match the definitions of ethnic groups between the Census categories and the homicide categories. Our challenge was that the classification of Asian ethnicity in the Census, and ONS data of homicide victims was different. In the ONS homicide victimisation data, Asian included only victims from the Indian subcontinent. In the census data, Asian included people from all of the continent of Asia. Our least-worst solution to this challenge was to match the definition across the two datasets by using the following classification: White (White British, White Irish, and White Gypsy and White other); Black (Black African, Black Caribbean, and Black other); Asian, Indian subcontinent (Pakistan, India, Bangladesh, Sri Lankan); and Other (Arabs; Chinese; Asian [other]; mixed; any other). Using these definitions appears to offer the most precise common boundaries possible around numerators and denominators.

## Methods

We also used similar methods to those in our previous analysis of England and Wales, but with greater emphasis on tracking the magnitude of disparity between ethnic groups in homicide victimisation rates over time. Here again, we did not estimate changing sizes of the population of each ethnic group considered. We applied the 2001 denominator up through 2010–2011 and then applied the 2011 denominator for all years thereafter.

All ratios between victimisation rates of Blacks, Whites and Asians were computed by simply dividing the group initially reporting a higher rate per 100,000 people by the rate of the group with the initially lower rate for each year.

## Findings

**Fig. 1:**
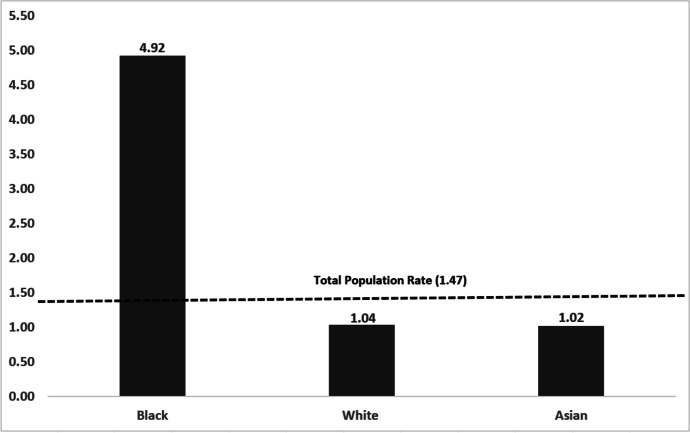
Five-year homicide victimisation rate (per 100,000) average by ethnicity London 2017–2022

Figure 1 shows the most recent differences in homicide victimisation (2017–2022) amongst the three largest ethnic groups in London. Inequality is most pronounced between Black victimisations and all other homicides. In the rest of these findings, we report comparisons of racial and demographic inequalities in the two largest pairs of groups in London: Black/White and Asian/White.

### Black-White Inequalities

#### 22-Year Trends

**Fig. 2:**
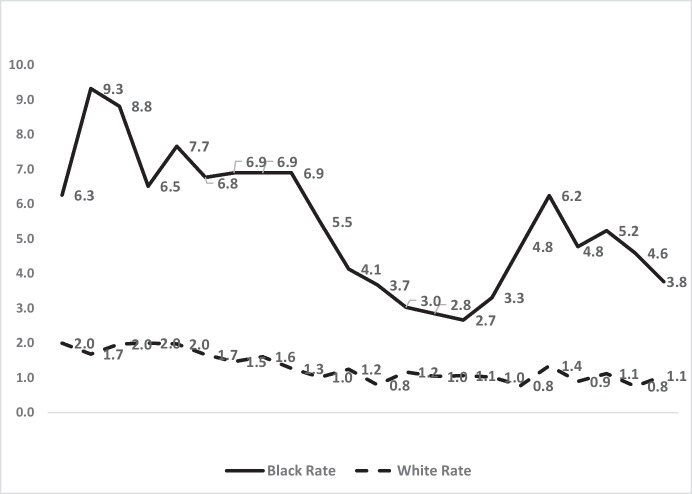
Homicides per 100,000 London residents for Blacks and Whites 2000–2022

Figure 2 reveals two major facts about Black homicide victimisation risk. One is that the homicide rates of Blacks in London were very high at the start of the century, and then dropped fairly steadily by 245% up to 2014/15—from 9.3 per 100,000 down to 2.7. The other fact is that this trend reversed from 2014/15 until 2017–2018, when it hit a second peak at 6.2 homicides per 100,000 Black residents.

This “fall and rise” pattern is mitigated by the most recent 3 years, in which Black homicide victimisations subsided back to levels a decade earlier. Yet the most recent year shows a victimisation rate that is still 45% higher than the lowest year in that decade. And compared to somewhat larger reductions in White rates, Black rates in the most recent 5 years were 19% more *disparate* from Whites in the last 5 years than in the first—an indication of increasing inequality.

#### Black-White Ratios

**Fig. 3:**
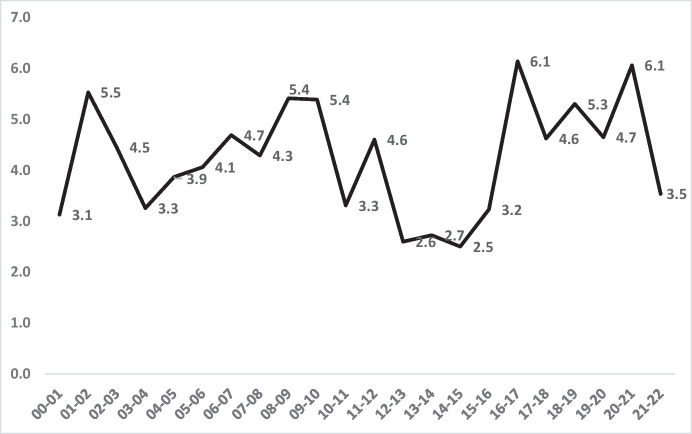
Annual ratio Black to White Homicide victimisation rates, London: 2000–2022

As an overall statement of racial equality, Fig. 2 offers a clarity of the magnitude of both the differences in the rates of the two populations and the changes within them over time. Yet it is not as clear as a single line showing the trend in the inequality ratio between Black and White rates. Figure 3 offers that single line, which is arguably the bellwether indicator of racial inequality in homicide victimisation.

To offer transparency in how the ratios in Fig. 3 were calculated for each year, Table 1 displays the exact rates of homicide victimisation within the two racial groups by year, and the ratio in each year between the homicide rates of the two largest groups (Blacks and Whites).

**Table 1:** Homicide rates per 100,000 Blacks and Whites in London 2000–2022 and the ratio by year

Year	Victimisation rate per 100,000: Black	Victimisation rate per 100,000: White	Black-to-White rate ratio of rates per 100,000
2000–2001	6.3	2.0	3.1
2001–2002	9.3	1.7	5.5
2002–2003	8.8	2.0	4.5
2003–2004	6.5	2.0	3.3
2004–2005	7.7	2.0	3.9
2005–2006	6.8	1.7	4.1
2006–2007	6.9	1.5	4.7
2007–2008	6.9	1.6	4.3
2008–2009	6.9	1.3	5.4
2009–2010	5.5	1.0	5.4
2010–2011	4.1	1.2	3.3
2011–2012	3.7	0.8	4.6
2012–2013	3.0	1.2	2.6
2013–2014	2.8	1.0	2.7
2014–2015	2.7	1.1	2.5
2015–2016	3.3	1.0	3.2
2016–2017	4.8	0.8	6.1
2017–2018	6.2	1.4	4.6
2018–2019	4.8	0.9	5.3
2019–2020	5.2	1.1	4.7
2020–2021	4.6	0.8	6.1
2021–2022	3.8	1.1	3.5

Figure 3 displays the data from Table 1 with the clearest indicator of the racial disparity in homicide victimisation over time: the *ratio between* the Black and White homicide victimisation rates. It differs from the trends in Fig. 2 because it is sensitive to changes in homicide rates of two races simultaneously. This means that Black victimisation can decline even as racial inequality increases. For example, as the Black victimisation *rate* went *down* in 2020–2021, the inequality *ratio* went *up* because the White rate dropped much faster than the Black that year. This is but one of many examples of the potential differences between reducing homicides *within* races and reducing inequality *between* races.

Figure 3 therefore shows a paradox, in combination with Fig. 2. Whilst we know that Black homicide victimisation was somewhat lower at the end of the 2 decades than at the start, the inequality *ratio* went up whilst the homicide rate went down. Rather than declining somewhat from start to finish (like the homicide rate), the inequality ratio was 19% higher in the last 5 years of the 2 decades (an average of 4.8 per 100,000) than in the first 5 years (an average of 4.06 per 100,000).

#### Highest Risk Group: Young Males Aged 16–24

**Fig. 4:**
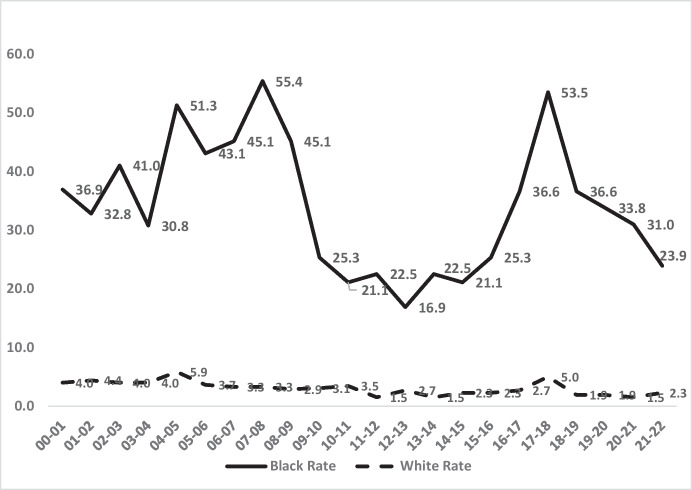
Male homicide victimisation rates for Blacks and Whites per 100,000 residents for age group 16–24, London, 2000–2022

In order to understand these trends, it is useful to focus on the demographic subset of the London population with the highest risk of homicide, as widely established in criminology: the subset of young males aged 16–24. The rates of homicide in that age group have been consistently much higher than for the general population in London as elsewhere (see Fig. 4). For Black men, homicide rates for age 16–24 have been as high as 30 times the general population rate (all races).

Figure 4 shows that the high risks for young males are also highly unequal and of much greater magnitude amongst young males than amongst the general population. The racial disparity in homicide victimisation rates within this group is far greater than in the population as a whole.

What is similar in comparing the young male subset to the general population’s data is the fall and rise of both homicide and inequality. Figure 4 shows the same points in time for major reductions in homicide rates for Blacks in London: 2007/2008 to 2014/2015 and again in 2017 through 2021/2022. Thus, there is a consistency of pattern for the fall and rise trends in homicides of all ages (Fig. 2) and of young males (Fig. 4), both Black and White.

**Fig. 5:**
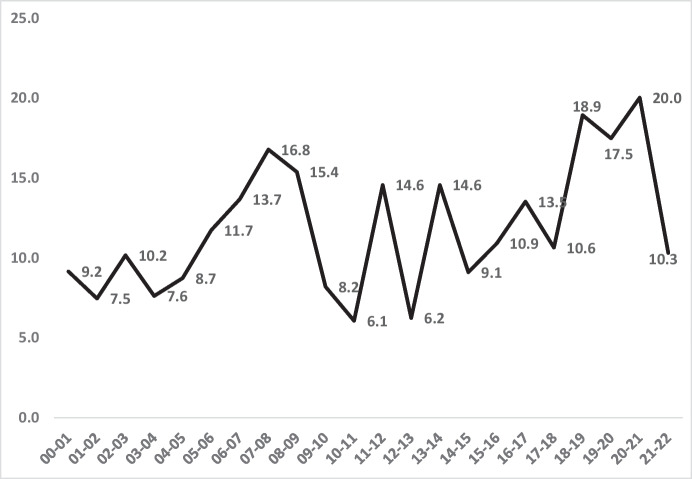
The annual ratio of homicide victimisation rates between Black and White males aged 16 to 24 in London, 2000–2022

The victimisation rates for young Black males are not, of course, the entire story of inequality in this age/gender category. The rates of White young male homicide deaths in London have remained low, but not flat. As Table 2 (and Fig. 5) shows, the precise ratio between the homicide rates per 100,000 of both Black and White males of that age has varied substantially from year to year. The range is from a low of 6.1 times higher homicide rates for Blacks than Whites in 2010/2011 to a high point of 20 times higher homicide rates for Blacks than Whites in 2020/2021.

**Table 2:** The ratio of Black to White homicide victimisation rates, males aged 16–24, London, 2000–2022

Year	Black rate	White rate	Black-to-White rate ratio
2000–2001	36.9	4.0	9.2
2001–2002	32.8	4.4	7.5
2002–2003	41.0	4.0	10.2
2003–2004	30.8	4.0	7.6
2004–2005	51.3	5.9	8.7
2005–2006	43.1	3.7	11.7
2006–2007	45.1	3.3	13.7
2007–2008	55.4	3.3	16.8
2008–2009	45.1	2.9	15.4
2009–2010	25.3	3.1	8.2
2010–2011	21.1	3.5	6.1
2011–2012	22.5	1.5	14.6
2012–2013	16.9	2.7	6.2
2013–2014	22.5	1.5	14.6
2014–2015	21.1	2.3	9.1
2015–2016	25.3	2.3	10.9
2016–2017	36.6	2.7	13.5
2017–2018	53.5	5.0	10.6
2018–2019	36.6	1.9	18.9
2019–2020	33.8	1.9	17.5
2020–2021	31.0	1.5	20.0
2021–2022	23.9	2.3	10.3

**Fig. 6:**
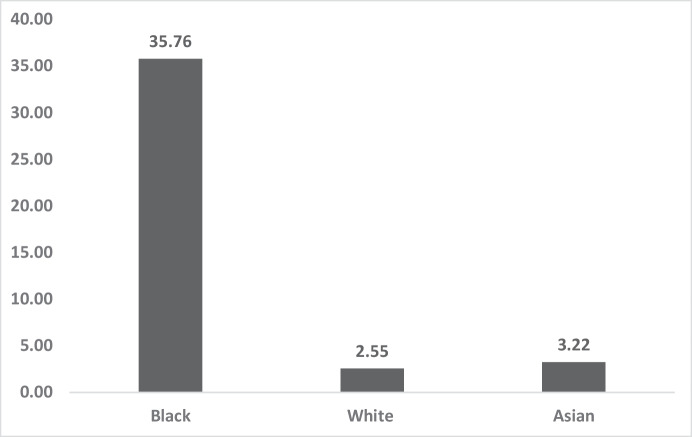
Male-only 5-year homicide victimisation rate (per 100,000) annual mean by ethnicity for age group 16–24, London, 2017–2022

Given smaller numbers of people in specific age and gender groups than across all people of all ages, there may be a chance for differences from year to year. This suggests that the fluctuation may be driven as much by “noise” as by “signal” (Kahneman et al., 2022)—i.e. chance fluctuations versus underlying real trends. One way to reduce “noise” is to compare 5-year periods, which can stabilise the results around a longer-term “signal” by filtering out short-term noise. Figure 6 therefore offers the average rates of the most recent 5-year period presented in Table 2’s year-on-year totals.

Figure 6 shows, again, the pattern of very similar homicide victimisation rates of Whites and Asians, whilst Black homicide victimisation rates are far higher. Whilst Asian males aged 16–24 had a homicide rate 26% higher than White males in that age group, Black males that age had a rate that was 1300% higher than that of Whites.

#### Black vs. White Female Victimisation Rates

**Fig. 7:**
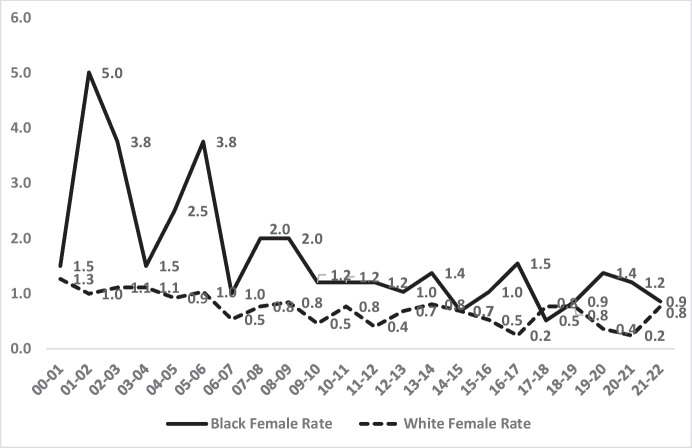
Black vs. White female victimisation rates per 100,000 by year 2000–2022

**Fig. 8:**
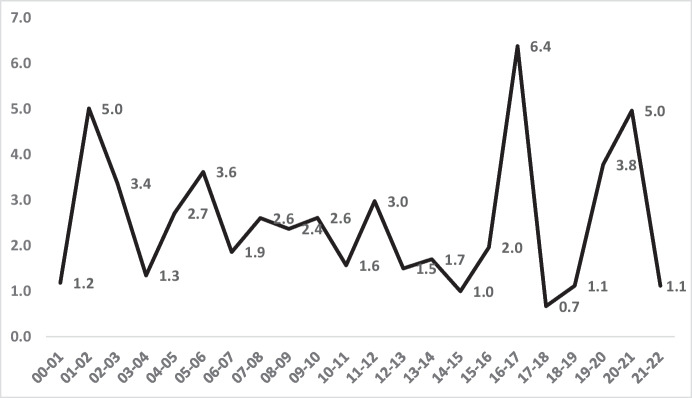
The Black vs. White female homicide victimisation rates ratio by year 2000–2022

From the highest risk group of males (age, 16–24) to a lower risk group (females of all ages), the continuing pattern of racial inequality is also evident across 2 decades. Figure 7 shows the declining rate of Black female homicide victimisations over the 2 decades, ending the period with much lower rates than at the outset. Yet Fig. 8 also shows the stability of inequality even as absolute rates of homicide decline. The final 5 years of the ratio, compared to the first 5 years, show somewhat higher disparity in rates (2.7 times higher) for Black females than for White females, compared to a somewhat lower average difference (2.3 times higher) in rates in the first 5 years. This difference constitutes an 18% increase of the magnitude of inequality in homicide victimisation rates of females of all ages in London.

#### Over Age 55: Black vs. White Homicide Inequality 2010–2022

**Fig. 9:**
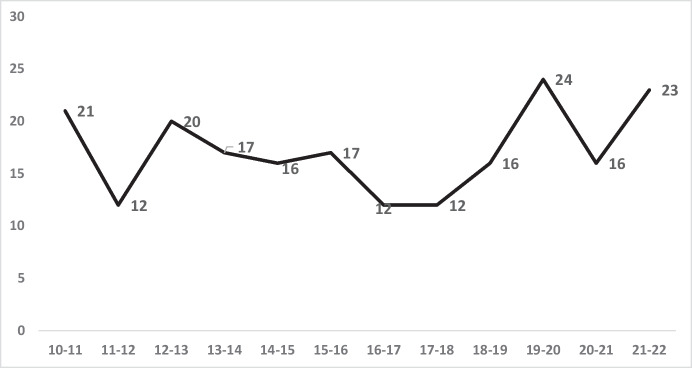
Total number of homicides in London for all persons over 55, 2010–2011 to 2021–2022

The vulnerability of older people makes them particularly concerned about crime, even though many studies show they have far lower victimisation rates for violent crimes than do people in younger age groups. Yet even with lower rates, older people may still suffer racial inequality in risk. In doing so, we found a fairly flat recent annual frequency of all homicides of people over age 55, with an average of 17.2 per year in 2010–2011 rising slightly to 18.2 per year in 2021–2022 (see Fig. 9).

**Fig. 10:**
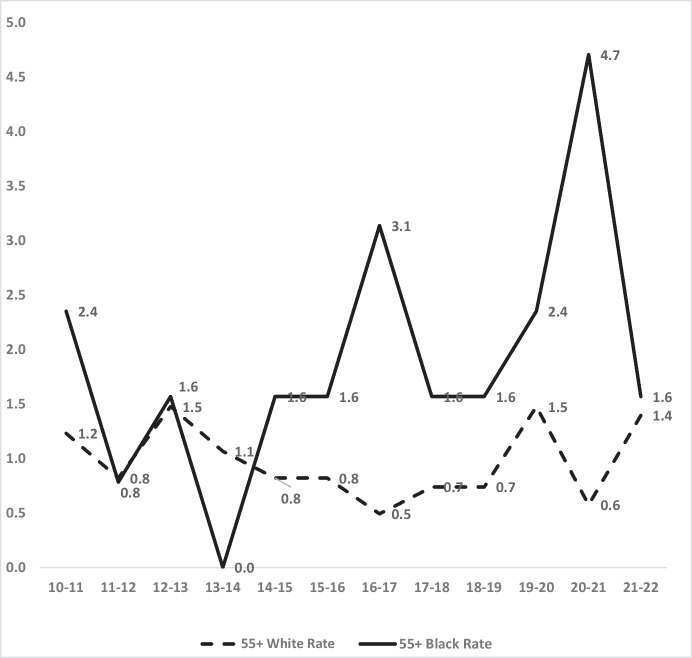
Black and White rates of homicide victimisations per 100,000 in London, 2010–2011 through 2021–2022

Figure 10 shows that even in this low-risk age 55 + group, inequality in homicide victimisation can be observed. Black homicide victimisation exceeds White rates amongst persons aged 55 or older in 9 of the 12 years described in Fig. 10, with the most recent 5 years showing an average ratio of 2.4 to 1—over twice as many homicides per capita of Blacks than Whites.

### Asian-White Comparisons

**Fig. 11:**
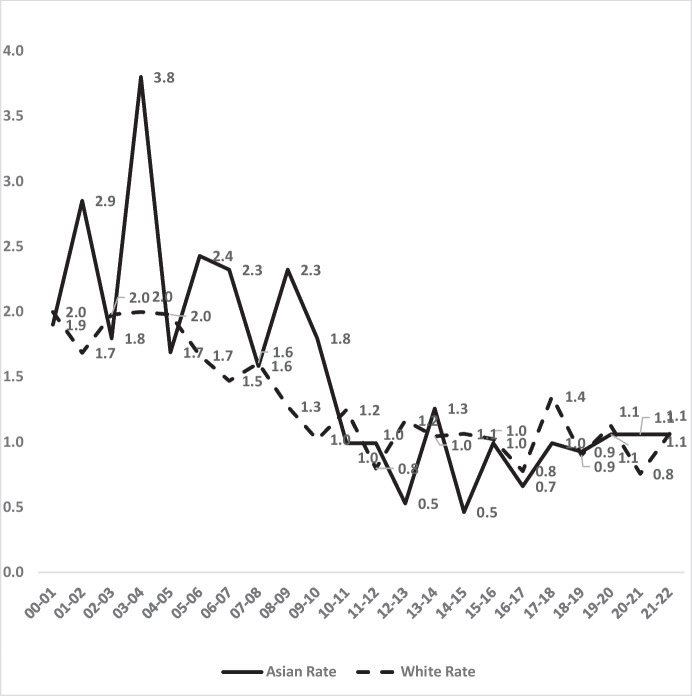
Asian vs. White homicide victimisations per 100,000 residents in London 2000–2022

Figure 11 shows very similar rates of homicide victimisations for Asians and Whites in London during 2000–2022. Yet even these rates began the century with somewhat higher homicide rates of Asians than of Whites; Asians peaked at 3.8 per 100,000 whilst Whites never exceeded 2.4. Both peaks occurred in the first decade.

**Fig. 12:**
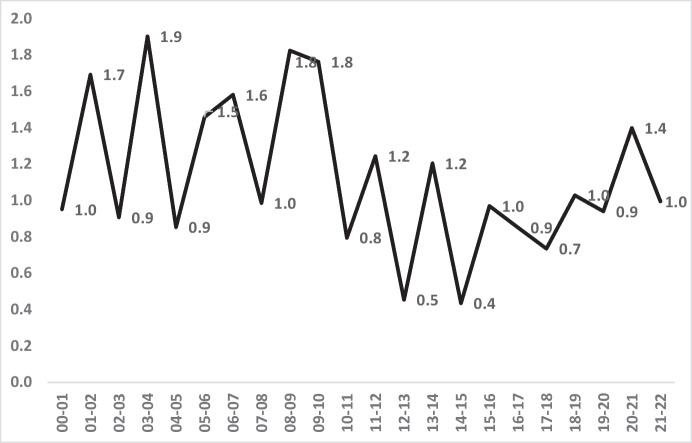
The ratio of Asian vs. White homicide victimisation rates per 100,000 by year in London, 2000–2022

By the second decade of the century, homicide rate inequality between Asians and Whites disappeared in London—at least at a macro-level that includes all residents. Figure 12 shows the trends in the annual ratio of homicide rates per 100,000 between Asians and Whites. In the most recent 5 years, the average annual ratio was 1.0, or one White homicide per 100,000 White residents for one Asian homicide per 100,000 Asians. This 1-to-1 ratio indicates zero inequality. Compared to the 1.26 to 1 Asian-to-White ratio in the first 5 years, the most recent ratio is a relative 22% reduction to zero in the difference in rates. Taken together, Figs. 11 and 12 show measurable reductions in both numbers of homicides and inequality of homicide risk—at least across all age groups and both genders taken together.

**Fig. 13:**
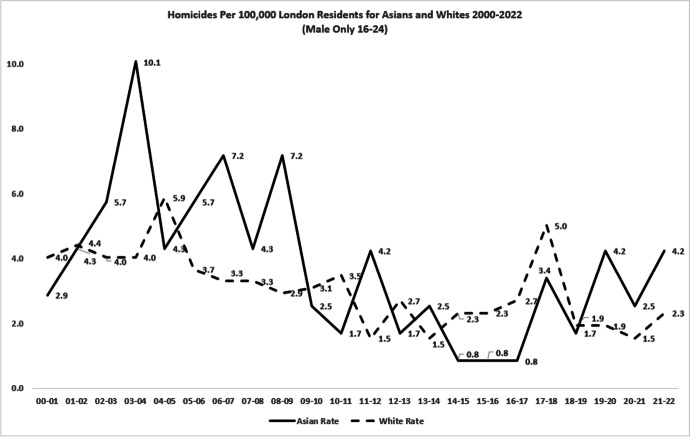
Annual homicide victimisation rates per 100,000 for Asian and White males aged 16–24, London, 2000–2022

**Fig. 14:**
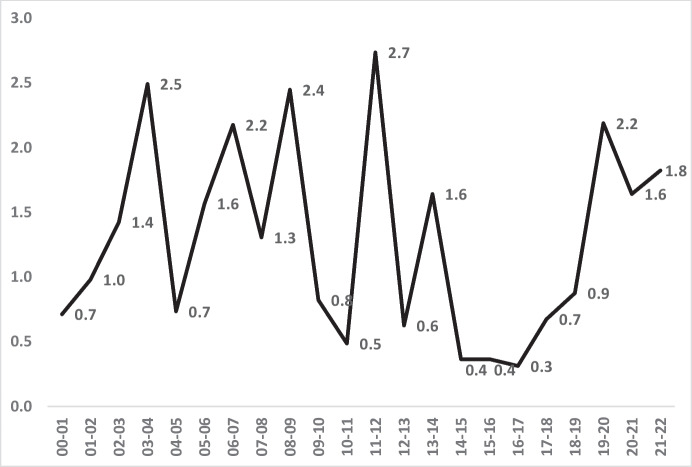
The annual ratio between Asian and White rates of homicide victimisation per 100,000 male residents aged 16–24

The disappearance of macro-level Asian-White inequality did not apply in all subgroups. As Figs. 13 and 14 show, both homicide rates and inequality were more intractable amongst males aged 16–24. Asian homicide victimisation rates in this young male subset peaked at 10.1 per 100,000 (Fig. 13), in comparison to the all-age/gender Asian peak of 3.8 per 100,000 (Fig. 11). The higher risk for young Asian men remained largely unchanged over 2 decades, but (as expected) remained much higher than for all Asians in London combined.

Figure 14 also shows that average annual young male inequality with White victimisation rates remained unchanged over 2 decades, despite the volatile changes from year to year. There was therefore less success with inequality amongst young males than for Asians in general. In the first 5 years of the century, homicide victimisation was 22% higher for Asian males 16–24 than for White males of that age. By the last 5 years, the difference remained virtually identical at 23% higher.

The magnitude of inequality for Asians also follows different patterns from the actual risk of victimisation. The contrast between all Asians vs. young male Asians may be due to a growth over time in low-risk demographic categories amongst all Asians; a greater proportion of women, or of older people, for example, could contribute to reduced inequality overall. Yet whatever the reasons, the White-Asian findings remained mixed when viewed strictly as a matter of inequality.

## Discussion

This article has revealed three major points of change in recent racial disparities of homicide rates in London: (1) a long drop in racial disparities in 2009–2015; (2) a sharp rise in disparities in 2015–2019; and (3) a modest reversal of that rise in the last 3 years. What can account for these three points of change?

Unfortunately, finding good answers to that question is well beyond the scope of the present report. What good answers require is much deeper analysis of many other facts, including the details of (1) each homicide case, and of (2) what quantity and kind of policing was being delivered in areas where homicides dropped or increased. Even the racial categories themselves must be re-examined, given London’s many varieties of both people labelled “White” (British and “other”) and “Black” (Caribbean, Nigerian, Somalian, etc.). The prevalence of guns vs. knives, geographic concentrations of falls and rises, times of day and days of the week—a wide range of dimensions could be examined to understand these trends.

### Retrospective vs. Prospective Analysis

Even with these additional details, there is much that we cannot tell from this kind of *retrospective* analysis. Absent controlled experimentation, there is no reliable way to determine what caused these trends to change over time. Any correlation between changes in policing and homicide rates, for example, might be explained away by similar correlations with other factors. But we could learn from these data to design *prospective* experiments in reducing both homicide and its inequalities.

### Suspect Characteristics

There is also a major issue of the characteristics of suspects or convicts in these murders. The extent to which suspects commit intra-racial vs. cross-racial homicide could vary over time, with suspect becoming more or less frequently linked to new criminal network structures. Numbers of suspects or accused participating in each murder, or in homicides of different kinds of victims, could also be important. How far from the homicide location or victim’s residence the suspects resided might change with trends up or down.

### Police Proactivity

Changes in the kind and volume of police intelligence about suspects prior to the homicides could also open new ways of understanding. Once suspects are identified, for example, they could be placed in a potential list of persons deemed likely to kill, based on prior serious violence or other factors developed from advanced data analytics (Berk et al., 2009).

### Data Sharing for Public Consent

In the short run, explanation does not need to be the main purpose of this kind of tracking. Public dialogue between police and community residents can be useful as soon as the tracking data are produced, regardless of whether they can be explained. Rather than keeping these data in the hands of professionals, displaying the data in each local community could share the burden of prevention between police and the public. Data of the kind presented in this report could be used to guide community consultations. Sharing the data with community residents and leaders could help them to consider alternative pathways for policing.

The decisions may be difficult, with not much certainty about what will work to reduce inequalities in the short run. That is all the more reason to enlist local residents in a community dialogue—one that may increase police legitimacy (Bottoms & Tankebe, 2012). Ironically, the most difficult decision may be to do *less policing* in most places, since most of London has no homicides at all over a decade (Jackson, 2010; Massey et al., 2019). Explaining to local elected officials why other parts of London have greater needs for police resources may be the one of the best possible uses of precision tracking of homicides.

### All Crime Types, Not Just Homicide

The uses of precise tracking for inequality are not limited to homicide. This report takes homicide as the leading indicator, a bellwether pointing to inequalities in other kinds of crime and deprivation. A sweep of many more offence types can be included in a comprehensive measure, such as the Cambridge Crime Harm Index (Sherman et al., 2016; Sherman et al., 2020), in order to compute racial inequality in two ways.

### City-Wide Index of Crime Inequality

One way is to develop a general index of crime inequality across all London, comparing each geographic unit to all others; a variety of units could be selected for this purpose and tracked on a monthly basis. The goal would be to drive down inequality in London’s crime, year on year.

### Local Rank Order in Public Safety

The other way is to assess each unit for its rank order position in Crime Harm Index totals per 1000 residents. Tracking inequality across London is important for everyone. Assigning resources where they are most needed is important for reducing inequality of crime harm by residential (or even commercial or recreational) locations. Whether the rank is calculated across 32 boroughs or 4835 Lower Super Output Areas (LSOAs), telling people where their community stands may be the first step towards a levelling up of information between police and the public.

## Conclusion

This report just scratches the surface of the potential for using more detailed evidence on the race and the risk to renew policing by consent. The data it presents is merely an invitation for closer examination, rather than the basis for any conclusive recommendations. The best use of the data presented here is not just to prove that inequality exists. Rather, the trend analysis indicates the kind of outcomes that police could address more directly. There is more to reducing crime than just the raw numbers. In a diverse community, inequality of victimisation must also be a part of every discussion of how to achieve less crime and more trust.

## Footnotes

1 Sources: London (Statista 2022), Madrid (Statista 2019 https://www.statista.com/statistics/980350/number-of-deaths-by-homicide-by-region-spain/) and New York (Wikipedia download 2022 https://en.wikipedia.org/wiki/Crime_in_New_York_City).
